# Crystal structure of bis­[4-(all­yloxy)-*N*′-(but-2-en-1-yl­idene)benzohydrazidato]nickel(II)

**DOI:** 10.1107/S2056989023002918

**Published:** 2023-04-14

**Authors:** Sultana Shakila Khan, Md. Belayet Hossain Howlader, Md. Chanmiya Sheikh, Ryuta Miyatake, Ennio Zangrando

**Affiliations:** a Department of Chemistry, Rajshahi University, Rajshahi-6205, Bangladesh; b Department of Applied Science, Faculty of Science, Okayama University of Science, Japan; c Center for Environmental Conservation and Research Safety, University of Toyama, 3190 Gofuku, Toyama, 930-8555, Japan; d Department of Chemical and Pharmaceutical Sciences, University of Trieste, Italy; University of Kentucky, USA

**Keywords:** crystal structure, nickel complex, all­yloxy, benzohydrazide

## Abstract

In the title complex, [Ni(C_14_H_15_N_2_O_2_)_2_], the nickel(II) atom exhibits a square-planar coordination geometry, being coordinated by two negatively charged *N*,*O* chelating ligands in a *trans* configuration, with the metal located on a crystallographic center of symmetry. The X-ray structural characterization showed the complex to be disordered over two orientations with refined occupancies of 0.898 (2) and 0.102 (2). The whole mol­ecule is close to planar, the five- and six-membered rings subtending a dihedral angle of 7.5 (2)°. The crystal packing is supported by C—H⋯π and C—H⋯O inter­actions that form a di-periodic layered network.

## Abstract 2

In the title mononuclear nickel(II) complex, the nickel(II) atom is bis­chelated by a carbohydrazinate ligand bearing unsaturated alkyl chains.

## 1. Chemical context

Hydrazones are a specific class of Schiff-base compounds that are distinguished by the presence of a –CO—NH—N= pharmacophore group, and exhibit a wide range of biological activity (Khan *et al.*, 2003 ▸; Joshi *et al.*, 2008 ▸; Terzioglu & Gürsoy, 2003 ▸). Hydrazone mol­ecules display a number of features, such as their degree of flexibility, a conjugated π-system and an NH unit that readily participates in hydrogen bonding and may be easily deprotonated. In addition, hydrazone mol­ecules behave as bidentate ligands through their carbonyl oxygen and azomethine nitro­gen atoms, and are widely used in coordination chemistry for their ability to form complexes with metal ions in variable oxidation states (Abou-Melha, 2021 ▸; Abser *et al.*, 2013 ▸; Saygıdeğer Demir *et al.*, 2021 ▸; Gond *et al.*, 2022 ▸; Velásquez *et al.*, 2020 ▸). In this respect, the formation of metal complexes plays an important role in enhancing the biological activity of hydrazones (Sathyadevi *et al.*, 2012 ▸). In addition, providing the mol­ecule with additional donor sites in this type of ligand can modulate the nuclearity of complexes (Vrdoljak *et al.*, 2023 ▸). As part of our studies in this area, this paper describes the crystal structure of a bis[benzo­hydrazidato]nickel(II) complex. 
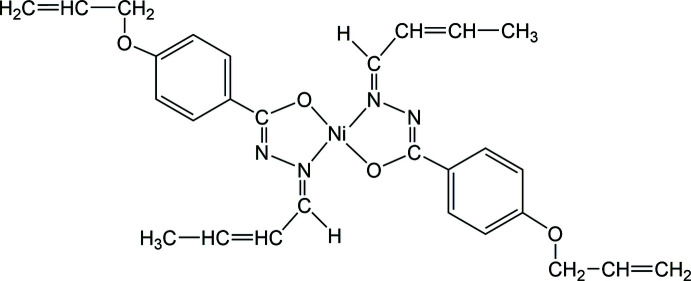


## 2. Structural commentary

**Figure 1:**
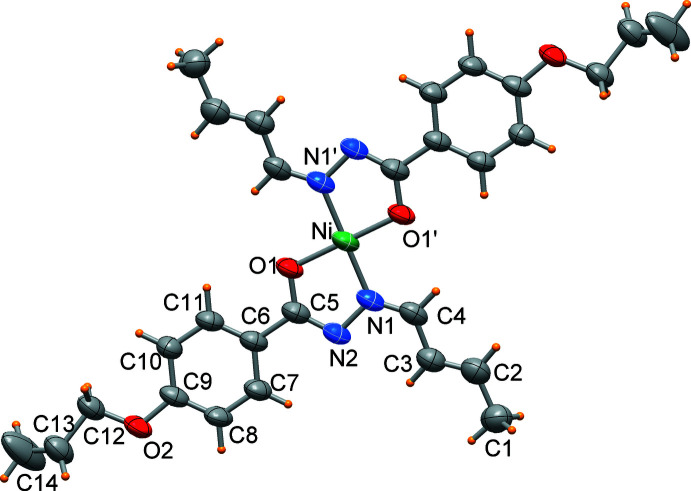
An ellipsoid plot (probability at 50%) of the Ni^II^ complex with atom labels for the crystallographically independent part.

**Figure 2:**
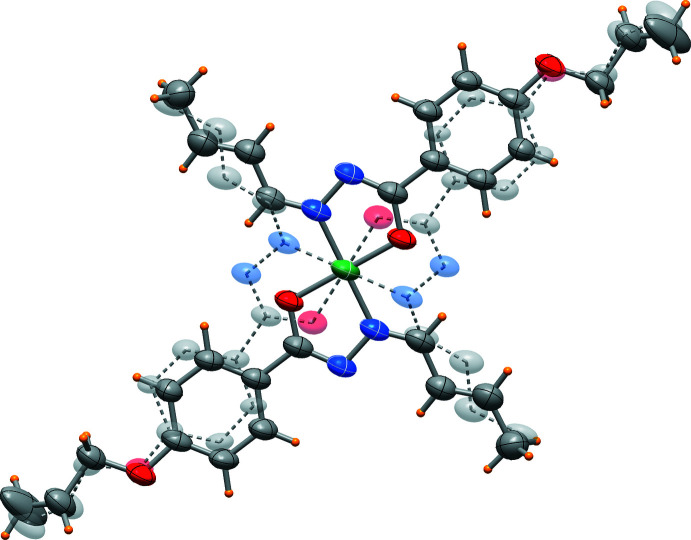
The two disordered species in the crystal with occupancies of *ca* 0.90/0.10.

The nickel(II) cation of the title complex, [Ni(C_14_H_15_N_2_O_2_)_2_], is located on a crystallographic inversion centre and exhibits a square-planar coordination geometry, with a *trans* configuration of the *N*,*O*-chelating ligands, as imposed by the crystal symmetry. An ellipsoid plot of the complex is shown Fig. 1 ▸. The structural characterization revealed that the complex is disordered over two orientations (Fig. 2 ▸) with refined occupancies of 0.898 (2) and 0.102 (2). As a result of the low percentage of the second component, the discussion is limited to the species at higher occupancy (Fig. 1 ▸). The Ni—O and Ni—N bond lengths are 1.8432 (16) and 1.8596 (18) Å, respectively, and the O1—Ni—N1 chelating angle is 84.13 (7)°. The C2—C3 and C13—C14 bond lengths are 1.319 (4) and 1.258 (5) Å, respectively, which confirm their double bond character (Allen *et al.*, 1987 ▸). Intra­molecular C4—H4⋯O1 and C11a—H11a⋯O1a inter­actions (Table 1 ▸), where the C⋯O distances are 2.975 (3) and 2.801 (3) Å, respectively, reinforce the crystal structure.

The X-ray diffraction analysis revealed that non-hydrogen atoms of the ligand are nearly coplanar; the maximum deviations being 0.308 (3) and 0.313 (5) Å for the allyl carbon atoms C13 and C14, respectively, on either side of the mol­ecular mean plane. The five- and six-membered rings form a dihedral angle of 7.5 (2)°. This conformation, which is rather common for this type of mol­ecule (Al-Qadsy *et al.*, 2021 ▸; Al Banna *et al.*, 2022 ▸; Krishnamoorthy *et al.*, 2012 ▸), allows for electron delocalization throughout the mol­ecule.

## 3. Supra­molecular features

**Figure 3:**
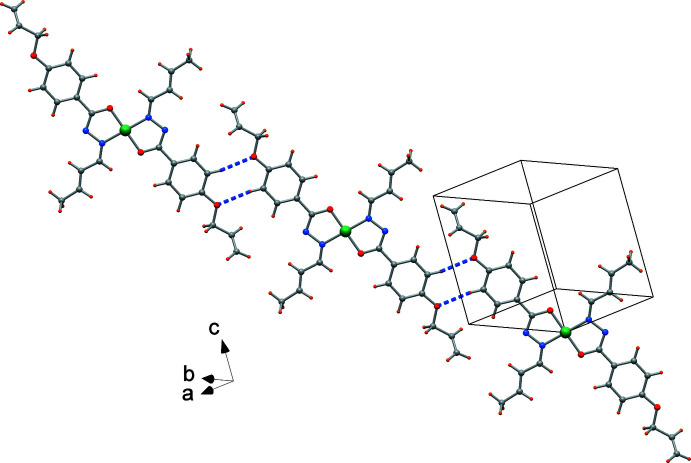
Mono-periodic chain formed by unconventional C—H⋯O hydrogen bonds (dotted lines) parallel to the [111] direction.

**Figure 4:**
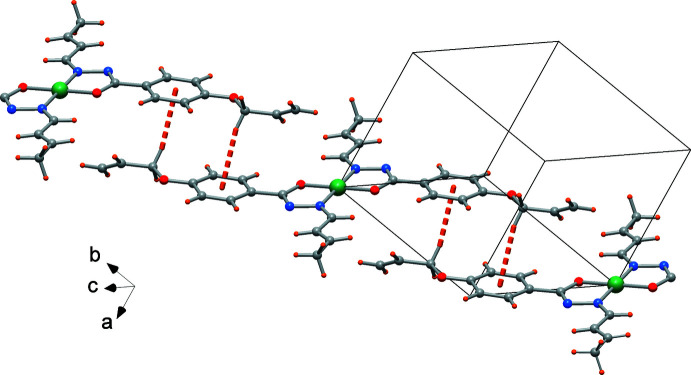
Detail of the crystal packing showing C—H⋯π inter­actions, forming a mono-periodic chain in the [011] direction.

**Figure 5:**
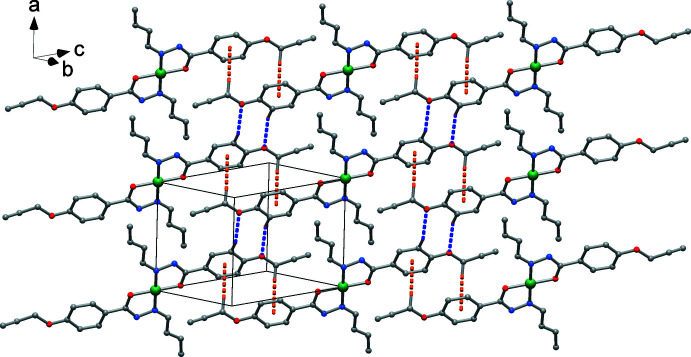
The di-periodic network built by C—H⋯O (blue dotted lines) and C—H⋯π (orange dotted lines) inter­actions. Only H atoms involved in the inter­actions are shown.

Despite the presence of phenyl rings in the ligands, there is no evidence of π–π stacking. The crystal packing is, however, supported by unconventional hydrogen bonds of type C—H⋯O, *e.g.* C8—H8⋯O2(−*x* + 1, −*y* + 1, −*z* + 1) that connect complexes to form ribbons in the [111] direction (Fig. 3 ▸, Table 1 ▸). In addition, C—H⋯π inter­actions are realized by centrosymmetrically related complexes (H⋯phenyl centroid distance = 2.88 Å, Table 1 ▸) and give rise to a polymeric chain in the crystallographic [011] direction (Fig. 4 ▸). These inter­actions form a di-periodic architecture, as depicted in Fig. 5 ▸.

## 4. Synthesis and crystallization

To a solution of 4-(all­yloxy)benzohydrazide (0.514 g, 2.6 mmol in 20 mL of ethanol), crotonaldehyde (0.187 g, 2.6 mmol) was added and the mixture was refluxed for an hour. Then a solution of nickel(II) acetate tetra­hydrate (0.335 g, 1.3 mmol in 10 mL of ethanol) was added and refluxing was continued for an additional two hours. The resulting orange precipitate was filtered off and washed with hot ethanol. The product was recrystallized from a mixture of chloro­form and toluene (1:1, *v*/*v*), and orange crystals, suitable for X-ray diffraction, were formed. Yield: 0.44 g, 60%, melting point: 511–513 K.

FT–IR (KBr), (cm^−1^): 1636 for ν(C=N—N=C) moiety. Absence of ν(N—H) and ν(C=O) bands. ^1^H NMR (CDCl_3_, 400 MHz), δ: 7.85 (*d*, 2×2H, *J* = 8.8 Hz, C-2, 6), 6.85 (*d*, 2×2H, *J* = 9.2 Hz, C-3, 5), 6.92 (*d*, 2×1H, *J* = 10 Hz, –CH=N,) , 6.41 (*m*, 2×1H, –CH=CH—CH_3_), 4.56 (*dt*, 2×1H, *J* = 5.2 Hz, =CH—CH_3_), 1.99 (*dd*, 2×3H, *J* = 6.8 Hz, 2.8 Hz, –CH_3_), 4.56 (*d*, 2×2H, *J* = 6.8 Hz, –OCH_2_), 5.42 (*dq*, 2×H_a_, *J* = 17.2 Hz, 3.2 Hz, =CH_2_), 5.30 (*dq*, 2×H_b_, *J* = 10.8 Hz, 3.2 Hz, =CH_2_) , 6.05 (*m*, 2×H_c_, –CH=CH_2_). HRMS (FAB) calculated for C_28_H_30_N_4_O_4_Ni, [*M* + H]^+^: 545.1692, found: 545.1693.

## 5. Refinement

Crystal data, data collection and structure refinement details are summarized in Table 2 ▸. The structure is disordered, having a second component with a low occupancy of about 10%. The whole component at lower occupancy was refined with DELU and RIGU restraints, with bond lengths restrained to those at higher occupancy by use of the instruction SAME (Sheldrick, 2015*b* ▸). The hydrogen atoms were included at idealized positions, using a riding model with fixed isotropic displacement parameters [C—H = 0.95–0.99 Å; *U*_iso_(H) = 1.2 or 1.5 *U*_eq_(C)].

## Supplementary Material

Crystal structure: contains datablock(s) I. DOI: 10.1107/S2056989023002918/pk2680sup1.cif

Structure factors: contains datablock(s) I. DOI: 10.1107/S2056989023002918/pk2680Isup2.hkl

CCDC reference: 2232075

Additional supporting information:  crystallographic information; 3D view; checkCIF report

## Figures and Tables

**Table 1:** Hydrogen-bond geometry (Å, °) *Cg*1 is the centroid of the C6–C11 ring.

*D*—H⋯*A*	*D*—H	H⋯*A*	*D*⋯*A*	*D*—H⋯*A*
C4—H4⋯O1^i^	0.95	2.46	2.975 (3)	114
C8—H8⋯O2^ii^	0.95	2.55	3.466 (5)	161
C11a—H11a⋯O1a	0.95	2.48	2.801 (3)	100
C12—H12b⋯*Cg*1^iii^	0.95	2.88	3.781 (4)	152

Symmetry codes: (i) 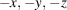 ; (ii) 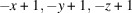 ; (iii) 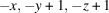 .

**Table 2:** Experimental details

Crystal data	
Chemical formula	[Ni(C_14_H_15_N_2_O_2_)_2_]
*M*_r_	545.27
Crystal system, space group	Triclinic, *P* 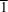
Temperature (K)	173
*a*, *b*, *c* (Å)	8.0978 (8), 9.2021 (9), 9.3316 (10)
α, β, γ (°)	84.027 (6), 88.091 (6), 84.170 (6)
*V* (Å^3^)	687.83 (12)
*Z*	1
Radiation type	Mo *K*α
μ (mm^−1^)	0.74
Crystal size (mm)	0.29 × 0.19 × 0.11

Data collection	
Diffractometer	Rigaku R-AXIS RAPID
Absorption correction	Multi-scan (*ABSCOR*; Higashi, 1995 ▸)
*T*_min_, *T*_max_	0.739, 0.988
No. of measured, independent and observed [*I* > 2σ(*I*)] reflections	6566, 3120, 2678
*R*_int_	0.027
(sin θ/λ)_max_ (Å^−1^)	0.649

Refinement	
*R*[*F* ^2^ > 2σ(*F* ^2^)], *wR*(*F* ^2^), *S*	0.040, 0.098, 1.03
No. of reflections	3120
No. of parameters	333
No. of restraints	196
H-atom treatment	H-atom parameters constrained
Δρ_max_, Δρ_min_ (e Å^−3^)	0.64, −0.20

Computer programs: *RAPID-AUTO* (Rigaku, 2018 ▸), *SHELXT2018/2* (Sheldrick, 2015*a* ▸), *SHELXL2019/2* (Sheldrick, 2015*b* ▸), *DIAMOND* (Brandenburg, 1999 ▸) and *WinGX* publication routines (Farrugia, 2012 ▸).

## supplementary crystallographic information

### Crystal data

**Table:**

[Ni(C_14_H_15_N_2_O_2_)_2_]	*Z* = 1
*M**_r_* = 545.27	*F*(000) = 286
Triclinic, *P*1	*D*_x_ = 1.316 Mg m^−^^3^
*a* = 8.0978 (8) Å	Mo *K*α radiation, λ = 0.71075 Å
*b* = 9.2021 (9) Å	Cell parameters from 8347 reflections
*c* = 9.3316 (10) Å	θ = 2.0–27.4°
α = 84.027 (6)°	µ = 0.74 mm^−^^1^
β = 88.091 (6)°	*T* = 173 K
γ = 84.170 (6)°	Prism, colorless
*V* = 687.83 (12) Å^3^	0.29 × 0.19 × 0.11 mm

### Data collection

**Table:**

Rigaku R-AXIS RAPID diffractometer	2678 reflections with *I* > 2σ(*I*)
Detector resolution: 10.000 pixels mm^-1^	*R*_int_ = 0.027
ω scans	θ_max_ = 27.5°, θ_min_ = 3.0°
Absorption correction: multi-scan (*ABSCOR*; Higashi, 1995)	*h* = −10→10
*T*_min_ = 0.739, *T*_max_ = 0.988	*k* = −11→10
6566 measured reflections	*l* = −12→12
3120 independent reflections	

### Refinement

**Table:**

Refinement on *F*^2^	196 restraints
Least-squares matrix: full	Hydrogen site location: inferred from neighbouring sites
*R*[*F*^2^ > 2σ(*F*^2^)] = 0.040	H-atom parameters constrained
*wR*(*F*^2^) = 0.098	*w* = 1/[σ^2^(*F*_o_^2^) + (0.0653*P*)^2^] where *P* = (*F*_o_^2^ + 2*F*_c_^2^)/3
*S* = 1.03	(Δ/σ)_max_ < 0.001
3120 reflections	Δρ_max_ = 0.64 e Å^−^^3^
333 parameters	Δρ_min_ = −0.20 e Å^−^^3^

### Special details

**Table:**

Geometry. All esds (except the esd in the dihedral angle between two l.s. planes) are estimated using the full covariance matrix. The cell esds are taken into account individually in the estimation of esds in distances, angles and torsion angles; correlations between esds in cell parameters are only used when they are defined by crystal symmetry. An approximate (isotropic) treatment of cell esds is used for estimating esds involving l.s. planes.

### Fractional atomic coordinates and isotropic or equivalent isotropic displacement parameters (Å^2^)

**Table:**

	*x*	*y*	*z*	*U*_iso_*/*U*_eq_	Occ. (<1)
Ni1	0.000000	0.000000	0.000000	0.05459 (14)	
O1	0.0243 (2)	0.07363 (16)	0.17356 (18)	0.0578 (4)	0.898 (2)
O2	0.2723 (5)	0.4718 (5)	0.6267 (4)	0.0674 (10)	0.898 (2)
N1	0.2013 (2)	0.07530 (17)	−0.04863 (19)	0.0579 (4)	0.898 (2)
N2	0.2596 (2)	0.15342 (17)	0.05752 (18)	0.0576 (4)	0.898 (2)
C1	0.6805 (6)	0.1843 (6)	−0.3689 (5)	0.0823 (13)	0.898 (2)
H1A	0.716800	0.236081	−0.290787	0.123*	0.898 (2)
H1B	0.663757	0.253188	−0.455829	0.123*	0.898 (2)
H1C	0.765440	0.104813	−0.388854	0.123*	0.898 (2)
C2	0.5204 (4)	0.1213 (3)	−0.3251 (3)	0.0697 (7)	0.898 (2)
H2	0.469034	0.073371	−0.394546	0.084*	0.898 (2)
C3	0.4453 (3)	0.1270 (2)	−0.1979 (2)	0.0630 (5)	0.898 (2)
H3	0.496134	0.171140	−0.125341	0.076*	0.898 (2)
C4	0.2889 (4)	0.0685 (4)	−0.1661 (4)	0.0568 (9)	0.898 (2)
H4	0.245553	0.019621	−0.239171	0.068*	0.898 (2)
C5	0.1563 (3)	0.1451 (2)	0.1675 (2)	0.0552 (5)	0.898 (2)
C6	0.1878 (3)	0.2237 (2)	0.2942 (2)	0.0541 (5)	0.898 (2)
C7	0.3212 (3)	0.3097 (2)	0.2903 (2)	0.0596 (5)	0.898 (2)
H7	0.395956	0.312765	0.209668	0.072*	0.898 (2)
C8	0.3441 (4)	0.3901 (3)	0.4036 (3)	0.0620 (6)	0.898 (2)
H8	0.434157	0.449102	0.399809	0.074*	0.898 (2)
C9	0.2377 (6)	0.3857 (6)	0.5226 (4)	0.0582 (9)	0.898 (2)
C10	0.1064 (4)	0.2975 (3)	0.5305 (3)	0.0568 (6)	0.898 (2)
H10	0.034480	0.291804	0.613008	0.068*	0.898 (2)
C11	0.0832 (3)	0.2176 (2)	0.4142 (3)	0.0570 (6)	0.898 (2)
H11	−0.006304	0.158031	0.417885	0.068*	0.898 (2)
C12	0.1649 (5)	0.4721 (5)	0.7530 (4)	0.0683 (10)	0.898 (2)
H12A	0.175044	0.374580	0.809471	0.082*	0.898 (2)
H12B	0.047605	0.497658	0.725636	0.082*	0.898 (2)
C13	0.2227 (4)	0.5876 (3)	0.8394 (3)	0.0822 (7)	0.898 (2)
H13	0.250302	0.677449	0.789198	0.099*	0.898 (2)
C14	0.2360 (9)	0.5703 (6)	0.9742 (5)	0.159 (3)	0.898 (2)
H14A	0.209241	0.481443	1.026860	0.190*	0.898 (2)
H14B	0.272886	0.646034	1.023245	0.190*	0.898 (2)
O1'	0.1078 (17)	0.0981 (16)	0.1178 (14)	0.061 (3)	0.102 (2)
O2'	0.262 (3)	0.459 (4)	0.634 (3)	0.052 (6)	0.102 (2)
N1'	−0.1507 (14)	−0.0071 (12)	0.1524 (15)	0.054 (3)	0.102 (2)
N2'	−0.1064 (15)	0.0573 (14)	0.2739 (15)	0.055 (3)	0.102 (2)
C1'	−0.664 (4)	−0.157 (3)	0.394 (3)	0.055 (5)	0.102 (2)
H1'1	−0.755231	−0.211867	0.369214	0.082*	0.102 (2)
H1'2	−0.605399	−0.210640	0.476523	0.082*	0.102 (2)
H1'3	−0.708624	−0.059756	0.418061	0.082*	0.102 (2)
C2'	−0.545 (2)	−0.142 (3)	0.267 (3)	0.066 (5)	0.102 (2)
H2'	−0.564913	−0.182154	0.179826	0.079*	0.102 (2)
C3'	−0.4083 (19)	−0.0693 (19)	0.278 (2)	0.064 (4)	0.102 (2)
H3'	−0.380715	−0.041637	0.368440	0.077*	0.102 (2)
C4'	−0.305 (2)	−0.034 (3)	0.152 (3)	0.048 (5)	0.102 (2)
H4'	−0.355132	−0.029991	0.061133	0.057*	0.102 (2)
C5'	0.0340 (17)	0.1135 (17)	0.2405 (17)	0.054 (3)	0.102 (2)
C6'	0.115 (3)	0.199 (2)	0.345 (2)	0.057 (5)	0.102 (2)
C7'	0.248 (2)	0.2777 (18)	0.3065 (17)	0.045 (3)	0.102 (2)
H7'	0.303354	0.273720	0.215557	0.054*	0.102 (2)
C8'	0.295 (3)	0.363 (3)	0.408 (2)	0.057 (5)	0.102 (2)
H8'	0.390159	0.415000	0.389954	0.069*	0.102 (2)
C9'	0.205 (5)	0.374 (5)	0.538 (3)	0.047 (5)	0.102 (2)
C10'	0.067 (3)	0.299 (3)	0.578 (3)	0.058 (6)	0.102 (2)
H10'	0.005157	0.306756	0.665513	0.070*	0.102 (2)
C11'	0.031 (2)	0.211 (2)	0.475 (2)	0.057 (5)	0.102 (2)
H11'	−0.059768	0.152927	0.495134	0.069*	0.102 (2)
C12'	0.181 (3)	0.489 (3)	0.766 (2)	0.043 (4)	0.102 (2)
H12C	0.123609	0.403449	0.807081	0.052*	0.102 (2)
H12D	0.096411	0.574476	0.749571	0.052*	0.102 (2)
C13'	0.310 (3)	0.522 (2)	0.872 (2)	0.067 (5)	0.102 (2)
H13'	0.419193	0.474347	0.874300	0.080*	0.102 (2)
C14'	0.260 (4)	0.618 (4)	0.956 (4)	0.090 (11)	0.102 (2)
H14C	0.149387	0.663448	0.950229	0.108*	0.102 (2)
H14D	0.333053	0.643923	1.024081	0.108*	0.102 (2)

### Atomic displacement parameters (Å^2^)

**Table:**

	*U*^11^	*U*^22^	*U*^33^	*U*^12^	*U*^13^	*U*^23^
Ni1	0.0642 (2)	0.04564 (18)	0.0584 (2)	−0.01815 (13)	−0.01564 (14)	−0.00945 (13)
O1	0.0649 (9)	0.0544 (8)	0.0601 (9)	−0.0232 (7)	−0.0120 (8)	−0.0138 (7)
O2	0.082 (2)	0.0662 (17)	0.0625 (12)	−0.0312 (13)	−0.0128 (11)	−0.0200 (11)
N1	0.0698 (10)	0.0464 (8)	0.0617 (10)	−0.0166 (7)	−0.0183 (8)	−0.0099 (7)
N2	0.0645 (9)	0.0519 (8)	0.0613 (10)	−0.0190 (7)	−0.0158 (8)	−0.0114 (7)
C1	0.078 (2)	0.075 (3)	0.095 (3)	−0.0150 (15)	0.004 (2)	−0.0096 (17)
C2	0.0775 (16)	0.0562 (13)	0.0769 (17)	−0.0096 (11)	−0.0073 (14)	−0.0095 (12)
C3	0.0672 (13)	0.0575 (11)	0.0676 (14)	−0.0134 (10)	−0.0136 (11)	−0.0111 (10)
C4	0.0702 (15)	0.041 (2)	0.0612 (15)	−0.0112 (14)	−0.0175 (12)	−0.0067 (13)
C5	0.0619 (11)	0.0455 (9)	0.0613 (12)	−0.0135 (8)	−0.0175 (10)	−0.0067 (8)
C6	0.0590 (13)	0.0477 (11)	0.0592 (12)	−0.0166 (10)	−0.0154 (9)	−0.0073 (9)
C7	0.0623 (13)	0.0613 (12)	0.0605 (12)	−0.0236 (10)	−0.0126 (10)	−0.0106 (9)
C8	0.0648 (16)	0.0640 (15)	0.0636 (13)	−0.0295 (11)	−0.0125 (11)	−0.0111 (10)
C9	0.065 (3)	0.0514 (15)	0.0631 (17)	−0.0172 (17)	−0.0226 (13)	−0.0110 (14)
C10	0.0567 (15)	0.0579 (12)	0.0596 (15)	−0.0159 (11)	−0.0068 (11)	−0.0125 (12)
C11	0.0588 (15)	0.0527 (11)	0.0640 (17)	−0.0199 (11)	−0.0138 (13)	−0.0089 (13)
C12	0.084 (2)	0.061 (2)	0.0657 (17)	−0.0187 (15)	−0.0127 (14)	−0.0189 (14)
C13	0.109 (2)	0.0719 (15)	0.0724 (16)	−0.0207 (15)	−0.0135 (14)	−0.0226 (13)
C14	0.294 (7)	0.114 (4)	0.083 (2)	−0.071 (4)	−0.069 (3)	−0.011 (2)
O1'	0.053 (6)	0.068 (8)	0.066 (6)	−0.017 (6)	−0.012 (5)	−0.012 (5)
O2'	0.033 (8)	0.057 (11)	0.070 (8)	−0.009 (7)	−0.009 (6)	−0.013 (7)
N1'	0.045 (5)	0.034 (5)	0.084 (7)	0.002 (4)	−0.020 (5)	−0.014 (5)
N2'	0.054 (5)	0.044 (6)	0.070 (7)	−0.012 (5)	−0.010 (4)	−0.010 (5)
C1'	0.053 (8)	0.030 (9)	0.083 (11)	−0.007 (7)	−0.029 (7)	−0.006 (8)
C2'	0.062 (8)	0.055 (10)	0.084 (12)	−0.010 (7)	−0.020 (8)	−0.010 (9)
C3'	0.055 (6)	0.051 (8)	0.089 (9)	−0.009 (6)	−0.022 (6)	−0.003 (7)
C4'	0.046 (6)	0.017 (10)	0.077 (9)	0.005 (5)	−0.034 (5)	0.007 (6)
C5'	0.054 (6)	0.042 (7)	0.068 (7)	−0.014 (5)	−0.016 (5)	−0.007 (5)
C6'	0.055 (8)	0.056 (9)	0.064 (8)	−0.028 (7)	0.000 (6)	−0.011 (7)
C7'	0.037 (7)	0.038 (7)	0.062 (7)	−0.018 (6)	−0.001 (5)	−0.006 (6)
C8'	0.050 (9)	0.056 (10)	0.070 (8)	−0.029 (7)	0.005 (6)	−0.011 (7)
C9'	0.034 (8)	0.048 (11)	0.060 (8)	−0.007 (7)	−0.005 (5)	−0.006 (7)
C10'	0.044 (9)	0.070 (11)	0.069 (9)	−0.033 (8)	0.007 (7)	−0.024 (8)
C11'	0.052 (9)	0.059 (9)	0.067 (8)	−0.022 (7)	0.000 (6)	−0.013 (7)
C12'	0.047 (8)	0.022 (8)	0.058 (8)	−0.001 (6)	−0.009 (6)	0.007 (6)
C13'	0.071 (10)	0.053 (9)	0.077 (9)	0.003 (7)	−0.027 (8)	−0.014 (8)
C14'	0.071 (12)	0.084 (19)	0.12 (2)	0.011 (13)	−0.038 (12)	−0.051 (18)

### Geometric parameters (Å, º)

**Table:**

Ni1—O1'^i^	1.793 (13)	C13—C14	1.258 (5)
Ni1—O1'	1.793 (13)	C13—H13	0.9500
Ni1—O1	1.8432 (16)	C14—H14A	0.9500
Ni1—O1^i^	1.8432 (16)	C14—H14B	0.9500
Ni1—N1'^i^	1.843 (14)	O1'—C5'	1.287 (15)
Ni1—N1'	1.843 (14)	O2'—C9'	1.363 (16)
Ni1—N1	1.8596 (18)	O2'—C12'	1.423 (17)
Ni1—N1^i^	1.8596 (18)	N1'—C4'	1.301 (17)
O1—C5	1.308 (2)	N1'—N2'	1.406 (14)
O2—C9	1.369 (3)	N2'—C5'	1.311 (14)
O2—C12	1.442 (4)	C1'—C2'	1.509 (18)
N1—C4	1.289 (4)	C1'—H1'1	0.9800
N1—N2	1.4034 (19)	C1'—H1'2	0.9800
N2—C5	1.304 (3)	C1'—H1'3	0.9800
C1—C2	1.501 (4)	C2'—C3'	1.360 (16)
C1—H1A	0.9800	C2'—H2'	0.9500
C1—H1B	0.9800	C3'—C4'	1.441 (19)
C1—H1C	0.9800	C3'—H3'	0.9500
C2—C3	1.319 (4)	C4'—H4'	0.9500
C2—H2	0.9500	C5'—C6'	1.519 (15)
C3—C4	1.436 (4)	C6'—C7'	1.368 (16)
C3—H3	0.9500	C6'—C11'	1.386 (17)
C4—H4	0.9500	C7'—C8'	1.381 (16)
C5—C6	1.490 (3)	C7'—H7'	0.9500
C6—C11	1.381 (4)	C8'—C9'	1.401 (17)
C6—C7	1.400 (3)	C8'—H8'	0.9500
C7—C8	1.379 (3)	C9'—C10'	1.397 (17)
C7—H7	0.9500	C10'—C11'	1.374 (16)
C8—C9	1.383 (4)	C10'—H10'	0.9500
C8—H8	0.9500	C11'—H11'	0.9500
C9—C10	1.397 (3)	C12'—C13'	1.534 (18)
C10—C11	1.401 (3)	C12'—H12C	0.9900
C10—H10	0.9500	C12'—H12D	0.9900
C11—H11	0.9500	C13'—C14'	1.263 (18)
C12—C13	1.518 (3)	C13'—H13'	0.9500
C12—H12A	0.9900	C14'—H14C	0.9500
C12—H12B	0.9900	C14'—H14D	0.9500
			
O1'^i^—Ni1—O1'	180.0	H12A—C12—H12B	108.8
O1—Ni1—O1^i^	180.00 (4)	C14—C13—C12	123.3 (4)
O1'^i^—Ni1—N1'^i^	82.3 (5)	C14—C13—H13	118.4
O1'—Ni1—N1'^i^	97.7 (5)	C12—C13—H13	118.4
O1—Ni1—N1'^i^	125.4 (3)	C13—C14—H14A	120.0
O1^i^—Ni1—N1'^i^	54.6 (3)	C13—C14—H14B	120.0
O1'^i^—Ni1—N1'	97.7 (5)	H14A—C14—H14B	120.0
O1'—Ni1—N1'	82.3 (5)	C5'—O1'—Ni1	114.5 (12)
N1'^i^—Ni1—N1'	180.0	C9'—O2'—C12'	125 (2)
O1—Ni1—N1	84.13 (7)	C4'—N1'—N2'	114.5 (17)
O1^i^—Ni1—N1	95.87 (7)	C4'—N1'—Ni1	128.3 (15)
O1—Ni1—N1^i^	95.87 (7)	N2'—N1'—Ni1	115.4 (8)
O1^i^—Ni1—N1^i^	84.13 (7)	C5'—N2'—N1'	106.7 (12)
N1—Ni1—N1^i^	180.0	C2'—C1'—H1'1	109.5
C5—O1—Ni1	109.78 (15)	C2'—C1'—H1'2	109.5
C9—O2—C12	117.6 (3)	H1'1—C1'—H1'2	109.5
C4—N1—N2	117.3 (2)	C2'—C1'—H1'3	109.5
C4—N1—Ni1	128.85 (17)	H1'1—C1'—H1'3	109.5
N2—N1—Ni1	113.84 (14)	H1'2—C1'—H1'3	109.5
C5—N2—N1	107.80 (16)	C3'—C2'—C1'	119 (2)
C2—C1—H1A	109.5	C3'—C2'—H2'	120.4
C2—C1—H1B	109.5	C1'—C2'—H2'	120.4
H1A—C1—H1B	109.5	C2'—C3'—C4'	120.4 (18)
C2—C1—H1C	109.5	C2'—C3'—H3'	119.8
H1A—C1—H1C	109.5	C4'—C3'—H3'	119.8
H1B—C1—H1C	109.5	N1'—C4'—C3'	126 (2)
C3—C2—C1	125.3 (3)	N1'—C4'—H4'	116.9
C3—C2—H2	117.4	C3'—C4'—H4'	116.9
C1—C2—H2	117.4	O1'—C5'—N2'	121.1 (14)
C2—C3—C4	121.9 (2)	O1'—C5'—C6'	117.6 (13)
C2—C3—H3	119.1	N2'—C5'—C6'	121.3 (13)
C4—C3—H3	119.1	C7'—C6'—C11'	121.5 (13)
N1—C4—C3	126.8 (3)	C7'—C6'—C5'	123.1 (15)
N1—C4—H4	116.6	C11'—C6'—C5'	114.7 (15)
C3—C4—H4	116.6	C6'—C7'—C8'	115.9 (13)
N2—C5—O1	124.2 (2)	C6'—C7'—H7'	122.1
N2—C5—C6	118.43 (19)	C8'—C7'—H7'	122.1
O1—C5—C6	117.4 (2)	C7'—C8'—C9'	121.2 (15)
C11—C6—C7	119.07 (18)	C7'—C8'—H8'	119.4
C11—C6—C5	120.9 (2)	C9'—C8'—H8'	119.4
C7—C6—C5	120.0 (2)	O2'—C9'—C10'	118.7 (18)
C8—C7—C6	120.0 (2)	O2'—C9'—C8'	117.2 (18)
C8—C7—H7	120.0	C10'—C9'—C8'	124.0 (16)
C6—C7—H7	120.0	C11'—C10'—C9'	112.0 (16)
C7—C8—C9	120.8 (2)	C11'—C10'—H10'	124.0
C7—C8—H8	119.6	C9'—C10'—H10'	124.0
C9—C8—H8	119.6	C10'—C11'—C6'	125.4 (15)
O2—C9—C8	115.2 (3)	C10'—C11'—H11'	117.3
O2—C9—C10	124.7 (3)	C6'—C11'—H11'	117.3
C8—C9—C10	120.2 (2)	O2'—C12'—C13'	109.1 (19)
C9—C10—C11	118.5 (2)	O2'—C12'—H12C	109.9
C9—C10—H10	120.7	C13'—C12'—H12C	109.9
C11—C10—H10	120.7	O2'—C12'—H12D	109.9
C6—C11—C10	121.4 (2)	C13'—C12'—H12D	109.9
C6—C11—H11	119.3	H12C—C12'—H12D	108.3
C10—C11—H11	119.3	C14'—C13'—C12'	115.3 (19)
O2—C12—C13	105.3 (3)	C14'—C13'—H13'	122.3
O2—C12—H12A	110.7	C12'—C13'—H13'	122.3
C13—C12—H12A	110.7	C13'—C14'—H14C	120.0
O2—C12—H12B	110.7	C13'—C14'—H14D	120.0
C13—C12—H12B	110.7	H14C—C14'—H14D	120.0
			
N1—Ni1—O1—C5	4.34 (13)	N1'^i^—Ni1—O1'—C5'	−179.7 (12)
N1^i^—Ni1—O1—C5	−175.66 (13)	N1'—Ni1—O1'—C5'	0.3 (12)
O1—Ni1—N1—C4	177.9 (3)	O1'^i^—Ni1—N1'—C4'	−18 (2)
O1^i^—Ni1—N1—C4	−2.1 (3)	O1'—Ni1—N1'—C4'	162 (2)
O1—Ni1—N1—N2	−4.24 (12)	O1'^i^—Ni1—N1'—N2'	178.1 (9)
O1^i^—Ni1—N1—N2	175.76 (12)	O1'—Ni1—N1'—N2'	−1.9 (9)
C4—N1—N2—C5	−178.9 (2)	C4'—N1'—N2'—C5'	−163.0 (18)
Ni1—N1—N2—C5	3.05 (18)	Ni1—N1'—N2'—C5'	3.0 (14)
C1—C2—C3—C4	177.7 (4)	C1'—C2'—C3'—C4'	−171 (2)
N2—N1—C4—C3	3.1 (5)	N2'—N1'—C4'—C3'	−30 (4)
Ni1—N1—C4—C3	−179.1 (2)	Ni1—N1'—C4'—C3'	165.8 (19)
C2—C3—C4—N1	−176.1 (3)	C2'—C3'—C4'—N1'	−158 (3)
N1—N2—C5—O1	0.8 (3)	Ni1—O1'—C5'—N2'	2 (2)
N1—N2—C5—C6	−178.15 (15)	Ni1—O1'—C5'—C6'	−178.4 (14)
Ni1—O1—C5—N2	−4.2 (2)	N1'—N2'—C5'—O1'	−3 (2)
Ni1—O1—C5—C6	174.79 (13)	N1'—N2'—C5'—C6'	177.0 (16)
N2—C5—C6—C11	−179.42 (19)	O1'—C5'—C6'—C7'	10 (3)
O1—C5—C6—C11	1.6 (3)	N2'—C5'—C6'—C7'	−169.9 (19)
N2—C5—C6—C7	3.0 (3)	O1'—C5'—C6'—C11'	−179 (2)
O1—C5—C6—C7	−175.96 (18)	N2'—C5'—C6'—C11'	1 (3)
C11—C6—C7—C8	−1.8 (3)	C11'—C6'—C7'—C8'	3 (4)
C5—C6—C7—C8	175.8 (2)	C5'—C6'—C7'—C8'	173 (2)
C6—C7—C8—C9	0.7 (5)	C6'—C7'—C8'—C9'	−4 (4)
C12—O2—C9—C8	179.7 (5)	C12'—O2'—C9'—C10'	−8 (7)
C12—O2—C9—C10	−0.5 (9)	C12'—O2'—C9'—C8'	176 (4)
C7—C8—C9—O2	−179.1 (4)	C7'—C8'—C9'—O2'	179 (4)
C7—C8—C9—C10	1.0 (7)	C7'—C8'—C9'—C10'	2 (7)
O2—C9—C10—C11	178.5 (5)	O2'—C9'—C10'—C11'	−176 (4)
C8—C9—C10—C11	−1.7 (7)	C8'—C9'—C10'—C11'	0 (6)
C7—C6—C11—C10	1.1 (3)	C9'—C10'—C11'—C6'	−2 (5)
C5—C6—C11—C10	−176.5 (2)	C7'—C6'—C11'—C10'	0 (5)
C9—C10—C11—C6	0.6 (5)	C5'—C6'—C11'—C10'	−171 (2)
C9—O2—C12—C13	−174.3 (5)	C9'—O2'—C12'—C13'	154 (4)
O2—C12—C13—C14	−138.1 (6)	O2'—C12'—C13'—C14'	145 (3)

Symmetry code: (i) −*x*, −*y*, −*z*.

### Hydrogen-bond geometry (Å, º)

*Cg*1 is the centroid of the C6–C11 ring.

**Table:**

*D*—H···*A*	*D*—H	H···*A*	*D*···*A*	*D*—H···*A*
C4—H4···O1^i^	0.95	2.46	2.975 (3)	114
C8—H8···O2^ii^	0.95	2.55	3.466 (5)	161
C11a—H11a···O1a	0.95	2.48	2.801 (3)	100
C12—H12b···*Cg*1^iii^	0.95	2.88	3.781 (4)	152

Symmetry codes: (i) −*x*, −*y*, −*z*; (ii) −*x*+1, −*y*+1, −*z*+1; (iii) −*x*, −*y*+1, −*z*+1.
